# A methodological framework to distinguish spectrum effects from spectrum biases and to assess diagnostic and screening test accuracy for patient populations: Application to the Papanicolaou cervical cancer smear test

**DOI:** 10.1186/1471-2288-8-7

**Published:** 2008-02-21

**Authors:** Caroline Elie, Joël Coste, the French Society of Clinical Cytology Study Group

**Affiliations:** 1 Department of Biostatistics, Groupe hospitalier Cochin – Saint Vincent de Paul and Université Paris-Descartes, Paris, France

## Abstract

**Background:**

A spectrum effect was defined as differences in the sensitivity or specificity of a diagnostic test according to the patient's characteristics or disease features. A spectrum effect can lead to a spectrum bias when subgroup variations in sensitivity or specificity also affect the likelihood ratios and thus post-test probabilities. We propose and illustrate a methodological framework to distinguish spectrum effects from spectrum biases.

**Methods:**

Data were collected for 1781 women having had a cervical smear test and colposcopy followed by biopsy if abnormalities were detected (the reference standard). Logistic models were constructed to evaluate both the sensitivity and specificity, and the likelihood ratios, of the test and to identify factors independently affecting the test's characteristics.

**Results:**

For both tests, human papillomavirus test, study setting and age affected sensitivity or specificity of the smear test (spectrum effect), but only human papillomavirus test and study setting modified the likelihood ratios (spectrum bias) for clinical reading, whereas only human papillomavirus test and age modified the likelihood ratios (spectrum bias) for "optimized" interpretation.

**Conclusion:**

Fitting sensitivity, specificity and likelihood ratios simultaneously allows the identification of covariates that independently affect diagnostic or screening test results and distinguishes spectrum effect from spectrum bias. We recommend this approach for the development of new tests, and for reporting test accuracy for different patient populations.

## Background

"Spectrum bias" in diagnostic test evaluation was first reported by Ransohoff and Feinstein in 1978 [1]. They observed that the sensitivity and specificity of diagnostic tests could differ between subgroups of patients with different characteristics, including severity and location of the disease or clinical features. Since this pioneering study, many authors have described such differences in performance for numerous tests in various contexts (e.g. [2-14]). It has been recommended that authors report estimates of variability of diagnostic accuracy between subgroups of patients affected by these differences in performance and this was recently endorsed by the STARD Initiative [15,16]. However, other authors have expressed scepticism regarding the evaluation of accuracy of diagnostic or screening tests, to the point of considering them "unpredictable" as their accuracy may depend on too many factors [17,18], and the use the post test probabilities (PTP) as indicators of test accuracy has been proposed [13].

As the literature became increasingly confused, the recent paper by Goehring et al. [19] represented an important breakthrough by drawing attention to the need for distinguishing between various "spectrum effects". Having defined "spectrum effect" as differences in the sensitivity or specificity of a diagnostic or screening test according to the patient's characteristics or to the features and severity of the disease, Goehring et al. showed that a "spectrum effect" can lead to a spectrum bias when subgroup variations in sensitivity or specificity also affect the likelihood ratios and thus post-test probabilities (see also [9,11,20]). Indeed, there are some situations for which subgroup analyses of sensitivity and specificity do not lead to the same conclusions as subgroup analyses for likelihood ratios. For example, conflicting results can be obtained when there is no variation in sensitivity and specificity between subgroups, but a higher prevalence of the disease in one subgroup than another. Conversely, variations in sensitivity and specificity do not mechanically imply biased results if one considers the "overall" test characteristics [19]. As sensitivity and specificity are inversely related, differences between subgroups do not necessarily affect likelihood ratios (and therefore post-test probabilities). Unfortunately, the term "bias" in "spectrum bias" may be misleading, as "bias" usually refers to the lack of validity of results due to inadequate study design (e.g. using a diagnostic case-control design to select patients rather than a diagnostic cohort design) and inadequate spectrum selection (e.g. by assessing an inappropriate group of patients given the study objective) [14,21]. Nevertheless we will conform to the work of Goehring et al. [19] and use this term herein because of its other classical meaning, which is statistical, regarding the use of an estimator giving wrong estimations: indeed, the post-test probabilities of diseases would be biased (and thus the clinical decision altered) if the appropriate group-specific performance values of the test are not used.

Goehring et al. [19] only proposed stratified analysis of spectrum effects and biases. The recent logistic regression approach by Janssens et al. [22] is complementary to that developed more than twenty years ago by Hlatky [2] and subsequently by Coughlin [23] and Moons [9] (among others) and extends this analysis to multivariable cases. Such multivariable analyses are necessary because factors responsible for differences in performance of tests are generally numerous and closely related.

Here, we propose a methodological framework, derived from the approaches described (both applied together for the first time), to distinguish spectrum effects from spectrum biases. Our purpose is to isolate factors independently affecting the diagnostic accuracy of a test. This approach is illustrated by an application to the Papanicolaou smear test for detection of cervical cancer.

## Methods

### Data sources

We undertook a secondary analysis of the study by the French Society of Clinical Cytology to compare the efficiency of the conventional Papanicolaou smear, ThinPrep liquid-based cytology and the Hybrid-Capture II human papillomavirus test (HPV test) [24-26]. The design of the study was described in detail elsewhere [24]. This analysis focuses on one of the three tests, the conventional Papanicolaou smear test, and the spectrum variations associated with it. All women included in this study (n = 1781) were evaluated by the reference standard (colposcopy followed by biopsy if abnormalities were detected), by the index test (conventional Papanicolaou smear test) and by the HPV test (which was considered in this analysis as a "spectrum" variable). These women were either referred for colposcopy because abnormalities had been detected on previous smears (referral clinic setting, n = 461) or were attending for routine smears (screening setting, n = 1320). Conventional Papanicolaou smear tests were read twice: in addition to routine reading in normal conditions ("clinical reading"), a reading blind to the context and clinical history was obtained for Papanicolaou test smears separately and independently by two different pathologists. In cases of disagreement, the slides were read again to reach a consensus conclusion, with a decision given, if necessary, by an independent expert ("optimized diagnosis"). Smear test results were classified as negative (normal smear or atypical squamous cells/glandular cells of undetermined significance (ASCUS/AGUS)) or positive (low grade or high grade squamous intraepithelial lesions or invasive cancer) according to the 1991 Bethesda system [27]. The reference standard results were classified as negative (normal colposcopy or negative biopsy result) or positive (cervical intraepithelial neoplasia of grade I, II or III or invasive carcinoma) according to the International Federation of Cervical Pathology and Colposcopy classification system [28]. The validity of these cutoff points may be open to discussion, but they were used in our previous papers and classify a sufficient number of patients with significant lesions. Other characteristics of the women were also recorded: age, current smoking, European or other origin, educational level, menopausal status and contraception status.

### Statistical analysis

Sensitivity, specificity and likelihood ratios were used as indicators of test accuracy. Stratified analyses of these indicators were performed for the following variables: HPV test, study setting (referral clinic or screening), age (< or ≥ 35 years), current smoking, European origin, educational level (higher education or less), menopausal status and contraception status (none, combined oral pill or other). Confidence intervals for sensitivity and specificity were produced with the Wilson score method without continuity correction [29]. Confidence intervals for positive and negative likelihood ratios were calculated by the method described by Simel et al. [30]. Logistic regression models were also constructed for sensitivity and specificity and the likelihood ratios to evaluate spectrum effects and spectrum biases associated with these variables.

The logistic model for sensitivity and specificity proposed by Coughlin et al. [23] was used to estimate sensitivity and specificity by defining the dependent variable as the dichotomous result of the diagnostic test. The presence of the disease defined by the reference standard is included as a binary explanatory variable, as are covariates potentially affecting sensitivity or specificity (Additional file 1). Interaction terms between the reference standard and covariates were also included to test whether the covariates affect sensitivity and specificity differentially.

The approach proposed by Janssens et al. [22] was used to estimate the likelihood ratios of a diagnostic test results conditional to covariates. It requires the construction of logistic models for the "prior odds" of the disease and one for "posterior odds" of the disease. The prior odds regression model includes only the covariate(s). The posterior odds regression model also includes the binary result of the diagnostic test and interaction terms between the diagnostic test and covariate(s), which indicate if the covariates affect the positive and the negative likelihood ratios differentially (Additional file 1). The likelihood ratios for the result of the diagnostic test conditional on the values of the covariates were further obtained by subtracting the coefficients of the prior odds regression model from the coefficients of the posterior odds regression model [22]. Confidence intervals for the differences in logistic regression coefficients were approximated by a bootstrap technique with 2000 random bootstrap samples with replacement [31].

All multivariable regression models initially included covariates modifying the test accuracy indicators with a p-value of 0.20 or less in logistic regression univariable analyses and first-order interactions between these covariates and the disease status (according to the diagnostic test or the reference standard). Final models were obtained by a grouped backward stepwise selection procedure. At each step, the variable with the least significant main effect was removed from the model if its interaction terms were associated with a p-value greater than 0.05. Even if not significant, all first-order interactions (for variables with a significant main effect) were conserved in the final model to obtain less biased estimations of group-specific likelihood ratios, as recommended by Janssens et al. [22].

All analyses were performed using SAS software version 8 [32].

## Results

Among the 1781 women included, 355 scored positive with the conventional Papanicolaou smear test (20%). Table 1 presents the characteristics of the 1781 women included and the results of stratified analysis of sensitivity, specificity and likelihood ratios. The smear test's accuracy differed substantially between subgroups, in particular for HPV test and study setting, both for clinical and optimized readings.

**Table 1:** Subgroup analysis of the sensitivity, specificity and likelihood ratios of the Papanicolaou smear test

			Clinical reading (n = 1781)				Optimized interpretation (n = 1777)			
			
Variables†	n (%)	Disease prevalence n (%)	Sensitivity %	Specificity %	Positive likelihood ratio	Negative likelihood ratio	Sensitivity %	Specificity %	Positive likelihood ratio	Negative likelihood ratio
	
HPV test										
Positive	537 (30)	282 (53)	**95**	**76**	**4.02**	**0.07**	**92**	**76**	**3.93**	**0.10**
Negative	1244 (70)	73 (6)	**64**	**93**	**9.31**	**0.38**	**64**	**93**	**8.85**	**0.38**
Study setting										
Screening	1320 (74)	70 (5)	**70**	**94**	**12.32**	**0.32**	**75**	**92**	8.96	0.27
Referral clinic	461 (26)	285 (62)	**93**	**60**	**2.34**	**0.12**	**89**	**77**	3.90	0.14
Age										
<35 years	981 (55)	158 (16)	89	**92**	**11.66**	0.12	83	**91**	9.44	0.19
≥ 35 years	800 (45)	197 (25)	88	**87**	**6.79**	0.14	89	**88**	7.37	0.12
Current smoking										
Yes	602 (34)	161 (27)	90	**88**	7.22	0.11	87	89	7.67	0.15
No	1161 (66)	188 (16)	87	**91**	9.98	0.14	87	90	8.94	0.15
European origin										
Yes	1623 (91)	312 (19)	88	**91**	**9.71**	0.13	87	90	**8.81**	0.15
No	158 (9)	43 (27)	91	**81**	**4.74**	0.11	83	86	**5.99**	0.19
Educational level										
< Higher	1063 (60)	227 (21)	88	**87**	**7.15**	0.14	87	**88**	7.31	0.15
≥ Higher	709 (40)	128 (18)	89	**93**	**14.38**	0.12	86	**92**	11.06	0.15
Postmenopausal										
Yes	174 (10)	44 (25)	86	88	7.49	0.15	93	91	10.09	0.08
No	1607 (90)	311 (19)	89	90	9.13	0.12	85	90	8.31	0.16
Contraception										
None	640 (36)	127 (20)	89	89	8.30	0.12	**87**	90	8.93	0.14
Combined oral pill	905 (51)	173 (19)	87	91	9.26	0.14	**83**	90	8.50	0.19
Others	236 (13)	55 (23)	91	91	9.68	0.10	**96**	87	7.27	0.04

† Data were missing for the following variables: current smoking (18 patients) and educational level (9 patients).

Significant comparisons are indicated in bold.

Table 2 provides a summary of univariable and multivariable results for sensitivity, specificity and likelihood ratios. For the sake of simplicity, this table reports only effects with p-values of less than 0.2. Several covariates modified sensitivity or specificity but few affected the likelihood ratio(s). The multivariable modelling allowed the number of covariates affecting diagnostic accuracy to be decreased by removing non-independent factors (current smoking, European origin or educational level) that were related to sensitivity and specificity or likelihood ratios through HPV test, age or study setting. For both clinical and optimized readings, HPV test, study setting and age affected specificity and sensitivity independently. For the clinical reading, HPV test and study setting were both responsible for a spectrum bias whereas age had no effect on likelihood ratios. For the optimized interpretation, the HPV test and age were the only two factors responsible for a spectrum bias.

**Table 2:** Summary of univariable and multivariable regression analyses for sensitivity/specificity and likelihood ratios of the Papanicolaou smear test (only p-values less than 0.20 are presented)

	Univariable analyses								Multivariable analyses							
		
	Clinical reading				Optimized interpretation				Clinical reading				Optimized interpretation			
				
	Sensitivity Specificity†		Likelihood ratios‡		Sensitivity Specificity†		Likelihood ratios‡		Sensitivity Specificity†		Likelihood ratios‡		Sensitivity Specificity†		Likelihood ratios‡	
								
Variables	Main effect	Interaction	Main effect	Interaction	Main effect	Interaction	Main effect	Interaction	Main effect	Interaction	Main effect	Interaction	Main effect	Interaction	Main effect	Interaction
HPV test	<0.001	0.03	<0.001	0.033	<0.001	0.18	<0.001	0.18	<0.001	0.09	<0.001	0.011	<0.001	0.15	<0.001	0.17
Study setting	<0.001	0.10	0.001	0.11	<0.001	-	0.023	-	<0.001	0.14	0.015	-	<0.001	-		
Age	0.001	0.06	-	0.07	0.04	-	0.12	-	0.026	-			0.045	-	0.047	-
									
Current smoking	0.03	-	-	-	-	-	-	-								
European origin	<0.001	-	-	-	0.17	-	-	-								
Educational level	<0.001	0.035	-	0.043	0.014	-	-	-								
Postmenopausal	-	-	-	-	-	0.17	-	-								
Contraceptive status	-	-	-	-	-	-	-	-								

HPV: human papillomavirus.

† For the sensitivity/specificity model: interaction between the variable and the reference standard (see Additional file 1)

‡ For the likelihood ratios model: interaction between the variable and the diagnostic test (see Additional file 1).

Additional files 2 and 3 contains details about the sensitivity, specificity (Additional file 2) and likelihood ratios (Additional file 3) of the final models for clinical reading.

## Discussion

We propose a methodological framework for identifying factors independently responsible for spectrum effects (i.e. which affect the sensitivity and specificity only) and for spectrum biases (i.e. which affect the likelihood ratios and post-test probabilities). This framework consists of double modelling, of sensitivity/specificity and positive/negative likelihood ratios respectively and therefore extends the stratified analysis of spectrum effects and biases proposed by Goehring et al. [19], taking into account the fact that these factors are generally numerous and closely related. We demonstrated the usefulness of this framework by application to Papanicolaou smear testing for the detection of cervical cancer. With this approach, we were able to differentiate the covariates linked to disease prevalence or severity and true "test modifiers" (modifying the test results due to their own effect, as HPV and age should do) from others factors affecting test accuracy only through "test modifiers" (for example current smoking, European origin and educational level). The massive and consistent effect of the HPV test result on Papanicolaou smear test results can be explained by the influence of the virus on cellular features. Disease prevalence and/or severity have a well-known effect on test accuracy indices [33]. Indeed, high risk (or oncogenic) HPV is the cause of cervical cancer development and is currently considered as a marker of severity of intraepithelial lesions [34,35]. The study setting was found to be responsible for spectrum bias only for clinical reading, confirming the information bias (or clinical review bias) observed for reading not blind to the context and clinical history. The strong effect of study setting probably masked the effect of age on the clinical reading, as age appeared to be responsible for spectrum bias only in optimized reading (where information bias was neutralized).

Many authors report differences in diagnostic or screening test accuracy between subgroups, but few have used a multivariable modelling approach to identify factors responsible for differences in the performance of tests and confounding factors. A review of current practice, including investigations of so-called spectrum bias (Table 3), shows that a large number of factors have been investigated, often without discernment but frequently with confusion regarding their significance to test accuracy. Moreover, most of these studies analyzed test accuracy only in terms of sensitivity and specificity [6-8,36-40], making it impossible to distinguish between spectrum effect and spectrum bias.

**Table 3:** Studies that investigated subgroup variations*

Study, Year	Approach to the analysis of spectrum bias/effect	Disease (number of subjects)	Diagnostic or screening test	Factors investigated	Effect on sensitivity and/or specificity	Effect on Likelihood ratios
Van der schouw, 1995 [5]	Subgroup analysis	Epididymitis (372)	Ultrasonography	Disease prevalence (severity according to clinician)	Disease prevalence (severity according to clinician)	Disease prevalence (severity according to clinician)
Morise, 1995 [36]	Subgroup analysis	Coronary disease (4467)	Exercise electrocardiography	Sex Verification by the gold standard (angiography) or not	Sex Verification bias by the gold standard (angiography) or not	Not considered
O'Connor, 1996 [6]	Subgroup analysis	Multiple sclerosis (303)	Magnetic resonance imaging and evoked potentials	Study group (two pooled studies considered), disease prevalence, clinical subjective disease probability	Study group, disease prevalence, clinical subjective disease probability	Not considered
Egglin, 1996 [37]	Subgroup analysis	Pulmonary embolism (24)	Pulmonary arteriogram	Disease prevalence	Disease prevalence	Not considered
Roger, 1997 [8]	Subgroup analysis	Coronary disease (3679)	Exercise echocardiography	Sex	Sex	Not considered
Curtin, 1997 [7]	Subgroup analysis	Obesity (226)	Body mass index	Weight, sex	Weight, sex	Not considered
Moons, 1997 [9]	Subgroup analysis and modelling of sensitivity	Coronary disease (295)	Exercise test	Patient history and clinical examination, various disease-related factors (maximal load, relative load, systolic blood pressure, number of diseased vessels)	Sex, expected load, maximal load, relative load, systolic blood pressure (baseline and peak), number of diseased vessels	Age, sex, symptoms, smoking, beta blocker use, cholesterol level, expected load, maximal load, relative load, systolic blood pressure (baseline and peak)
Santana-Bodao, 1998 [38]	Subgroup analysis	Coronary disease (702)	Single-photon emission computed tomography	Sex	Sex	Not considered
Steinbauer, 1998 [10]	Subgroup analysis	Alcohol abuse (1333)	Various screening tests	Race and sex	Race and sex	Race and sex
DiMatteo, 2001 [39]	Subgroup analysis	Group A beta haemolytic streptococcal pharyngitis (498)	Rapid antigen detection test	Disease severity	Disease severity	Not considered
Filly, 2002 [48]	Subgroup analysis and modelling	Cirrhosis (100)	Nodularity of surfaces of the liver	Deep versus superficial surfaces	Deep versus superficial surfaces	Age, sex, pathological type of cirrhosis
Hall, 2004 [40]	Subgroup analysis and modelling	Group A beta haemolytic streptococcal pharyngitis (561)	Rapid antigen detection test	Disease severity	Disease severity	Not considered
Meideros, 2005 [49]	Subgroup analysis	Glaucoma (136)	Scanning laser polarimetry	Two forms of glaucomatous optic neuropathy	Two forms of glaucomatous optic neuropathy	Two forms of glaucomatous optic neuropathy

* Papers published between 1978 and 2000 were identified from the systematic review by Whiting et al [14]. Papers published between 2000 and 2005 were similarly selected through Medline^® ^using the keywords: *diagnostic, test, screening, performance, accuracy, sensitivity, specificity, likelihood ratio, spectrum, subgroup, bias, prevalence, accuracy*. Only primary studies investigating variations in diagnostic test performances between subgroups were considered. Letters were excluded.

Our framework nevertheless presents some difficulties, mainly due to having to use non-trivial regression modelling. In particular, the simultaneous fitting of prior and posterior odds of the disease could be considered complex, as could the use of bootstrapping methods to construct confidence intervals for coefficients. Another difficulty is the management of interaction terms and the risk of colinearity between covariates included in the models. We chose to include only first order interactions between covariates and the disease status (according to the diagnostic test or the reference standard) because these interactions were the only ones relevant in the context of the differences in performance of a diagnostic or screening test. Usual recommendations concerning the practical implementation of regression analysis methods remain helpful in this context [41,42]. In particular, attention must be paid to the lack of power of the interaction test and its interpretation: the logistic model for sensitivity and specificity includes diseased and non-diseased patients and gives results closer to the sensitivity when the proportion of non-diseased patients is high, as is the case here. For example, we observe "paradoxical" results for current smoking, which is a significant predictor of sensitivity and specificity in the univariable analysis for clinical reading (Table 2, the interaction term is not significant), but with confidence intervals inconsistent with this conclusion. However, the use of a multivariable approach does not negate recommendations about patient selection or eliminate the necessity for carefully defined and relevant inclusion criteria – a spectrum of patients needs to be included that is similar to the population in which the test will be used in practice [15,16,43].

## Conclusion

In conclusion, we have shown the value of complementary and simultaneous modelling of sensitivity, specificity and likelihood ratios in logistic regression models: this approach can identify covariates that independently affect the accuracy of a diagnostic or screening test and can distinguish spectrum bias from spectrum effects. This approach appears preferable to subgroup analyses, which are classically recommended [15,16] but for which the problems are well known [44]: the number of patients per group is often small, especially if the number of covariates is high, leading to analyses that are not very powerful or accurate and problems of interpretation. As in therapeutic research [45-47], approaches based on regression modelling (and interaction testing) should replace subgroup analysis for the development of diagnostic and screening tests and for reporting their accuracy.

## Competing interests

The author(s) declare that they have no competing interests.

## Authors' contributions

JC conceived the study and its design, obtained funding source, provided study material, and administrative, technical, or logistic support. CE performed the statistical analysis and drafted the manuscript. All authors read and approved the final manuscript.

## Pre-publication history

The pre-publication history for this paper can be accessed here:



## Supplementary Material

Additional File 1 Logistic regression models for sensitivity, specificity and likelihood ratios of tests. The table presents Coughlin et al.'s model for sensitivity and specificity and Janssens et al.'s model for likelihood ratios (computation of indices, signification of main effects and signification of interactions). Click here for file

Additional File 2 Clinical reading: final multivariable regression model for sensitivity and specificity. The table presents the coefficients of the final regression model (Coughlin et al.'s model) and the method to calculate sensitivity and specificity from these coefficients. Click here for file

Additional File 3 Clinical reading: final multivariable regression model for the likelihood ratios. The table presents the coefficients of the final regression models (Janssens et al.'s models) and the method to calculate likelihood ratios from these coefficients. Click here for file
