# Supplementary material for: A methodological framework to distinguish spectrum effects from spectrum biases and to assess diagnostic and screening test accuracy for patient populations: Application to the Papanicolaou cervical cancer smear test
Source: BMC Med Res Methodol. 2008 Feb 21;8:7. doi: 10.1186/1471-2288-8-7 (PMC2291065; doi:10.1186/1471-2288-8-7)
Supplement: Additional File 1 — Logistic regression models for sensitivity, specificity and likelihood ratios of tests. The table presents Coughlin et al.'s model for sensitivity and specificity and Janssens et al.'s model for likelihood ratios (computation of indices, signification of main effects and signification of interactions). [file 1471-2288-8-7-S1.doc]

## Additional file 1: Logistic regression models for sensitivity, specificity and likelihood ratios of tests.

| Coughlin et al.’s model for sensitivity and specificity |  | Janssens et al.’s model for likelihood ratios |
| --- | --- | --- |
| Model equations | | |
| $\ln\left(\frac{P\left(T=1\right)}{1-P\left(T=1\right)}\right)=\gamma_{0}+\sum_{j=1}^{J} \gamma_{j}X_{j}+\gamma_{r}R$ |  | $\ln\left(prior odds\right)=\ln\left(\frac{p_{prior}}{1-p_{prior}}\right)=\alpha_{0}+\sum_{j=1}^{J} \alpha_{j}X_{j}$ $\ln\left(posterior odds\right)=\ln\left(\frac{p_{post}}{1-p_{post}}\right)=\beta_{0}+\sum_{j=1}^{J} \beta_{j}X_{j}+\beta_{t}T$ |
| Computation of indices |  |  |
| $Se=\frac{1}{1+\exp\left[-\left(\gamma_{0}+\sum_{j=1}^{J} \gamma_{j}X_{j}+\gamma_{r}R\right)\right]}$ where $R$=1 and the remaining covariates are assigned values corresponding to the subgroup of interest $Sp=1-\left\{\frac{1}{1+\exp\left[-\left(\gamma_{0}+\sum_{j=1}^{J} \gamma_{j}X_{j}+\gamma_{r}R\right)\right]}\right\}$ where $R$=0 and the remaining covariates are assigned values corresponding to the subgroup of interest |  | $LR+=\exp\left[\left(\beta_{0}-\alpha_{0}\right)+\sum_{j=1}^{J} \left(\beta_{j}-\alpha_{j}\right)X_{j}+\beta_{t}T\right]$ where $T$=1 to obtain the $LR+$ (and the remaining covariates are assigned values corresponding to the subgroup of interest) $LR-=\exp\left[\left(\beta_{0}-\alpha_{0}\right)+\sum_{j=1}^{J} \left(\beta_{j}-\alpha_{j}\right)X_{j}+\beta_{t}T\right]$ where $T$=0 to obtain the $LR-$ (and the remaining covariates are assigned values corresponding to the subgroup of interest)  because $posterior odds=\left(LR+\right)\times prior odds$ $\ln\left(LR\right)=\ln\left(posterior odds\right)-\ln\left(prior odds\right)$ and $\ln\left(LR/X_{J}\right)=\left(\beta_{0}-\alpha_{0}\right)+\sum_{j=1}^{J} \left(\beta_{j}-\alpha_{j}\right)X_{j}+\beta_{t}T$ |
| Signification of main effects |  |  |
| Effect of the covariate on $Se$ or $Sp$ |  | Effect of the covariate on $LR+$ or $LR-$ |
| Signification of interactions |  |  |
| Interaction between the covariate and $R$ indicates if the covariate affects $Se$ and $Sp$ differently |  | Interaction between the covariate and $T$ indicates if the covariate affects $LR+$ and $LR-$ differently |
| Notations | | |
| $T$ Disease status according to the diagnostic or screening test (0: not diseased, 1: diseased) $R$ Disease status according to the reference standard (0: not diseased, 1: diseased) $X_{j}$ Covariates ($j=1,...,J$) potentially modifying the accuracy of the test $prior odds$ Probability of disease before test, i.e. $p_{prior}=P\left(R=1\right)$ divided by $1-p_{prior}=P\left(R=0\right)$ $posterior odds$ Probability of disease after test, i.e. $p_{post}=P\left(R=1/X_{j}\right)$ divided by $1-p_{post}=P\left(R=1/X_{j}\right)$ $Se$ Sensitivity $Se=P\left(T=1/R=1\right)$ $Sp$ Specificity $Sp=P\left(T=0/R=0\right)$ $LR+$ Positive likelihood ratio $LR+=Se/\left(1-Sp\right)$ $LR-$ Negative likelihood ratio $LR-=\left(1-Se\right)/Sp$ | | |
