# Supplementary material for: A methodological framework to distinguish spectrum effects from spectrum biases and to assess diagnostic and screening test accuracy for patient populations: Application to the Papanicolaou cervical cancer smear test
Source: BMC Med Res Methodol. 2008 Feb 21;8:7. doi: 10.1186/1471-2288-8-7 (PMC2291065; doi:10.1186/1471-2288-8-7)
Supplement: Additional File 2 — Clinical reading: final multivariable regression model for sensitivity and specificity. The table presents the coefficients of the final regression model (Coughlin et al.'s model) and the method to calculate sensitivity and specificity from these coefficients. [file 1471-2288-8-7-S2.doc]

**Additional file 2: Clinical reading: final multivariable regression model for sensitivity and specificity**

| Variables | Regression coefficients  (95% CI) | | P value |
| --- | --- | --- | --- |
| Intercept | -3.41 | (-3.77 to -3.05) | <0.001 |
| Reference standard (positive) | 2.94 | (2.04 to 3.85) | <0.001 |
| HPV test (positive) | 1.48 | (1.07 to 1.90) | <0.001 |
| Study setting (referral clinic) | 2.31 | (1.90 to 2.72) | <0.001 |
| Age (≥ 35 years) | 0.45 | (0.05 to 0.85) | 0.0261 |
| Reference standard by HPV interaction | 0.74 | (-0.12 to 1.59) | 0.09 |
| Reference standard by study setting interaction | -0.65 | (-1.51 to 0.21) | 0.14 |
| Reference standard by age interaction | -0.51 | (-1.37 to 0.34) | 0.24 |

HPV: human papillomavirus

Sensitivity and specificity are calculated from these coefficients (see Appendix 1).

For example, in the group of women older than 35 years ($X_{age}=1$) and referred for colposcopy **(**$X_{study setting}=1$), the sensitivity ($R=1$) of the Papanicolaou smear test is equal to 1 / (1+exp[-(-3.41+2.94*1+1.48*1+2.31*1+0.45*1+0.74*1-0.65*1-0.51*1)])=0.97 if the HPV test is positive ($X_{HPV}=1$), whereas it is equal to 1 / (1+exp[-(-3.41+2.94*1+1.48*0+2.31*1+0.45*1+0.74*0-0.65*1-0.51*1)])=0.76 if the HPV test is negative ($X_{HPV}=0$).
