# Supplementary material for: A methodological framework to distinguish spectrum effects from spectrum biases and to assess diagnostic and screening test accuracy for patient populations: Application to the Papanicolaou cervical cancer smear test
Source: BMC Med Res Methodol. 2008 Feb 21;8:7. doi: 10.1186/1471-2288-8-7 (PMC2291065; doi:10.1186/1471-2288-8-7)
Supplement: Additional File 3 — Clinical reading: final multivariable regression model for the likelihood ratios. The table presents the coefficients of the final regression models (Janssens et al.'s models) and the method to calculate likelihood ratios from these coefficients. [file 1471-2288-8-7-S3.doc]

**Additional file 3: Clinical reading: final multivariable regression model for the likelihood ratios**

|  | Ln(post odds) | | | Ln(prior odds) | | | Ln(LR) | | |
| --- | --- | --- | --- | --- | --- | --- | --- | --- | --- |
| Variables | Regression coefficients  (95% CI) | | P value | Regression coefficients  (95% CI) | | P value | Computed coefficients†  (95% CI) | | P value |
| Intercept | -4.26 | (-4.75 to -3.78) | <0.001 | -3.92 | (-4.27 to -3.58) | <0.001 | -0.34 | (-0.71 to 0.03) | 0.071 |
| HPV test (positive) | 1.03 | (0.35 to 1.71) | 0.003 | 2.49 | (2.15 to 2.83) | <0.001 | -1.46 | (-2.08 to -0.84) | <0.001 |
| Study setting (referral clinic) | 2.29 | (1.64 to 2.94) | <0.001 | 3.03 | (2.68 to 3.37) | <0.001 | -0.73 | (-1.32 to -0.14) | 0.015 |
| Diagnostic test (positive) | 2.39 | (1.64 to 3.13) | <0.001 |  |  |  | 2.39 | (1.59 to 3.18) | <0.001 |
| Interaction between diagnostic test and HPV | 1.12 | (0.27 to 1.97) | 0.01 |  |  |  | 1.12 | (0.25 to 1.96) | 0.011 |
| Interaction between diagnostic test and study setting | -0.46 | (-1.29 to 0.37) | 0.28 |  |  |  | -0.46 | (-1.33 to 0.41) | 0.30 |

HPV: human papillomavirus, LR: likelihood ratio

† Coefficients of the ln(LR) model were calculated by subtracting the coefficients of the prior odds regression model from the coefficients of the posterior odds regression model (see Appendix 1).

Likelihood ratios are calculated from these coefficients (see Appendix 1). For example, in the group of women referred for colposcopy ($X_{study setting}=1$), the likelihood ratio of a positive Papanicolaou smear test ($T=1$) is equal to exp[-0.34-1.46*1-0.73*1+2.39*1+1.12*1-0.46*1]=1.68 if the HPV test is positive ($X_{HPV}=1$), whereas it is equal to exp[-0.34-1.46*0-0.73*1+2.39*1+1.12*0-0.46*1]=2.36 if the HPV test is negative ($X_{HPV}=0$).
